# Successful Management of Complex Rhegmatogenous Retinal Detachment in Congenital Glaucoma

**DOI:** 10.7759/cureus.19437

**Published:** 2021-11-10

**Authors:** Suzie Gasparian, Moises Enghelberg, Kakarla V Chalam

**Affiliations:** 1 Ophthalmology, Loma Linda University School of Medicine, Loma Linda, USA; 2 Retina Service, Loma Linda University Eye Institute, Loma Linda, USA

**Keywords:** hypotony maculopathy, silicone oil, retinal detachment, pars plana vitrectomy (ppv), congenital glaucoma

## Abstract

* *Retinal detachment in congenital glaucoma is rare and often associated with a poor prognosis. In this report, we describe ocular manifestations of congenital glaucoma, pre- and post-operative ophthalmic findings, and overall anatomic and functional outcomes after successful rhegmatogenous retinal detachment repair along with a review of the literature. Rhegmatogenous retinal detachment in a 45-year-old monocular patient with congenital glaucoma was successfully repaired with small gauge pars plana vitrectomy, intra-operative perfluorocarbon use and 1,000 centistoke silicone oil tamponade. Best-corrected visual acuity improved from CF to 20/70; however, the post-operative course was complicated by hypotony-associated maculopathy after removal of silicone oil. Five thousand centistoke silicone oil was reinfused with good anatomic and functional outcomes. The functional outcome may ultimately be limited by pre-existing amblyopia and other ocular comorbidities.

## Introduction

Primary congenital glaucoma is a rare, visually impairing disease with an incidence of roughly one in 10,000 live births [1]. It is characterized by abnormal development of the trabecular meshwork, aqueous outflow tracts of the eye, and consequential elevation of intraocular pressure [2,3]. The cornea and sclera are notably distended (enlarged corneal diameter and buphthalmos), with a subsequent increase in axial length [4,5]. Resultant myopia and increased refractive error predispose to retinal thinning and retinal detachment (RD) [6].

In the presence of other ocular comorbidities in congenital glaucoma, detection of retinal detachment is generally delayed, which increases the risk of poor anatomic and functional outcomes. Congenital glaucoma complicated by retinal detachment is rare and surgical outcomes are poor. Literature on the characteristic features of retinal pathology and detailed surgical management of retinal detachment in this subset of the patient population is limited.

In this report, we describe the unique course and clinical characteristics of retinal detachment associated with congenital glaucoma and detail the surgical approach, challenges, and long-term management strategy along with a detailed review of the literature.

## Case presentation

A 45-year-old male with a past medical history of congenital glaucoma (diagnosed at age 3) presented with decreased vision in the left eye. Past surgical history of the right eye included tube shunt placement at age 5, and explantation of an exposed tube shunt with simultaneous cyclophotocoagulation at age 20 for management of intraocular pressure. Repeat Ahmed valve re-implantation three years ago (for poorly controlled IOP) led to corneal decompensation. The patient underwent repeat Descemet’s Stripping Automated Endothelial Keratoplasty (DSAEK) in the right eye two years later with ensuing graft failure.

Past surgical history of the left eye was significant for Ahmed valve implantation for poorly controlled IOP (age 12), DSAEK for corneal opacity at a young age (age 16) along with cataract surgery seven years ago. Of note, the patient had a history of amblyopia along with severe glaucomatous optic nerve damage in the left eye with best-corrected visual acuity (BCVA) of 20/200.

**Figure 1:**
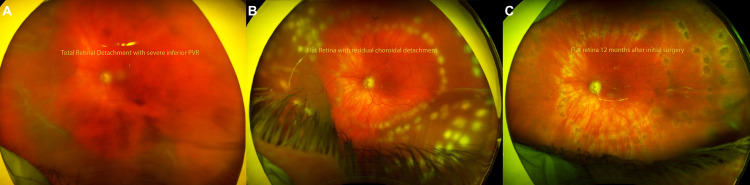
Retinal detachment repair (RRD) in congenital glaucoma. Ultrawidefield fundus photography (Optos, Marlborough, MA) demonstrates (A) a large nasal/inferior retinal detachment with associated proliferative vitreoretinopathy. Note the poor quality of the photo given limited visualization from a hazy corneal graft. (B) Post-operative day 1 after RRD repair with silicone oil tamponade with evidence of successful retinal attachment with the revelation of pre-existing underlying choroidal effusions. (C) Post-RRD repair with silicone oil tamponade with the successful anatomic outcome.

Upon presentation to the surgical retina service, BCVA was hand motion (HM) in the right eye (a hazy corneal graft) and CF at 1’ in the left eye. Intraocular pressure was 10 mmHg in the right eye and 8 mmHg in the left eye with maximal glaucoma therapy (dorzolamide, timolol, brimonidine, and latanoprost in both eyes). External examination demonstrated buphthalmic eyes bilaterally (axial length OD 28.20 mm and OS 29.76 mm and white-to-white values of 13.0 mm and 13.6 mm, respectively). Anterior segment examination was remarkable for DSAEK with central corneal scarring in the right eye, Haab’s striae with DSAEK in the left eye, and posterior chamber intraocular lens in each eye. Anterior vitreous examination revealed diffuse pigment dispersion (positive Shafer’s sign) in the left eye. Fundus examination of the left eye demonstrated a posterior vitreous detachment, an enlarged cup/disc (0.9) ratio, and severe choroidal effusion masked by a large nasal/inferior retinal detachment and associated inferior proliferative vitreoretinopathy (PVR) grade C along with multiple retinal tears inferiorly in the left eye (Figure 1A).

The patient underwent standard 23-gauge pars plana vitrectomy (PPV) for retinal detachment repair with careful dissection of proliferative membranes and drainage of subretinal fluid from pre-existing retinal breaks with the use of perfluorocarbon liquid. Endolaser was applied 360 degrees along the vitreous base and to areas of retinal breaks with subsequent instillation of 1,000 centistoke silicone oil. All glaucoma medications were discontinued after surgery. Post-operatively, the retina remained attached with improvement in BCVA to 20/100 and gradual resolution of choroidal effusions (Figure 1B). Silicone oil was removed four months later (patient request in view of monocular status) to improve BCVA.

**Figure 2:**
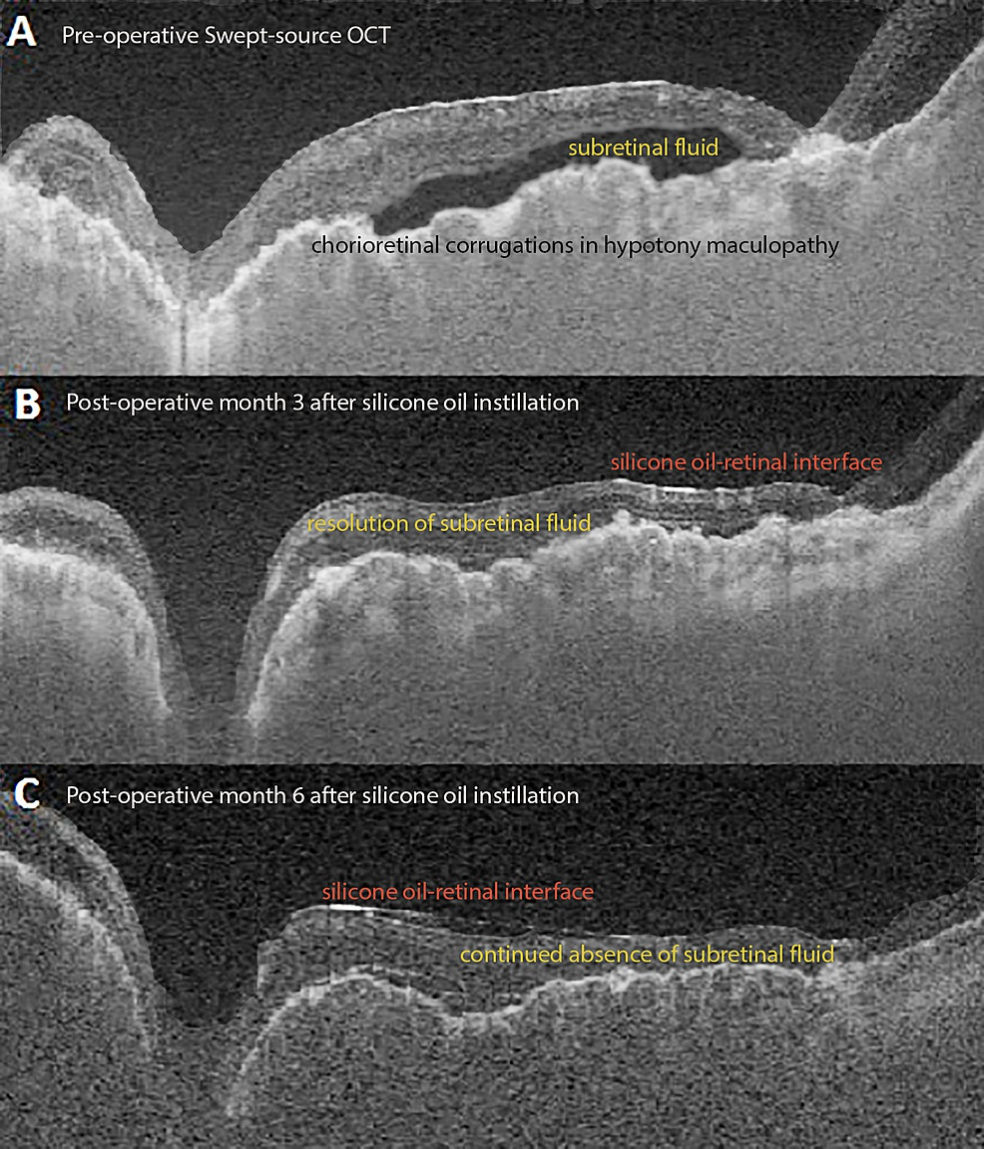
Hypotony maculopathy in congenital glaucoma. Swept-source ocular coherence tomography (Optos, Marlborough, MA) demonstrates (A) subtle chorioretinal corrugations and subretinal fluid consistent with hypotony maculopathy with (B) subsequent resolution after re-instillation of silicone oil at three months. (C) Sustained resolution of hypotony maculopathy 12 months after the placement of silicone oil.

One month after silicone oil removal, the patient developed hypotony-associated maculopathy (intraocular pressure of 4 mmHg) with an associated decrease in vision to HM (Figure 2A). At that point, Ahmed tube shunt was ligated. In view of persistent low intraocular pressure (IOP of 5 mmHg) and risk of phthisis bulbi despite successful anatomic outcome after RRD repair, the decision was made to place 5,000 centistoke silicone oil.

After silicone oil infusion, BCVA in OS improved to 20/200 with an increase in intraocular pressure to 13 mmHg and a successfully attached retina under silicone oil tamponade (Figures 1C, 2B, 2C). Twelve months after placement of silicone oil, BCVA in the left eye had improved to 20/70.

 

## Discussion

Reports of effective surgical outcome after retinal detachment with PVR in congenital glaucoma is limited. We describe the successful management of a monocular young male with a history of congenital glaucoma who presented with a long-standing retinal detachment associated with PVR. Retinal detachment was treated with PPV with intra-operative perfluorocarbon use and long-term silicone oil tamponade. Despite a promising anatomical result, visual outcome (20/70) was limited by prior history of amblyopia in the operated eye.

The average prevalence of buphthalmos is one in 30,000 births [7]. The buphthalmic eye consists of a visibly enlarged globe detected at or soon after birth secondary to uncontrolled intraocular pressure in early childhood. The largest increase in axial length occurs during the first few years of life. Elevated intraocular pressures induce an increase in axial length and corneal diameter in large part due to the elasticity of the infantile globe; this causes axial myopia and enlargement of the corneal diameter with ensuing thinning and breaks in Descemet’s membrane (Haab’s striae), a classic finding in buphthalmos.

The incidence of retinal detachment in buphthalmic eyes remains high. Studies note a higher incidence of peripheral retinal degeneration in buphthalmic eyes, especially eyes with axial length ≥26 mm [8]. Induced axial myopia coupled with increased peripheral retinal abnormalities predisposes buphthalmic eyes to retinal detachment [6]. Satofuka et al. additionally reported the presence of a posterior vitreous detachment due to advanced vitreous liquefaction in highly myopic eyes promotes the development of rhegmatogenous retinal detachment [9]. Knapp also considered degenerative changes within the vitreous implicit in the development of retinal detachment [10]. Wiedemann et al. considered retinal detachment to be a consequence of glaucoma filtering surgery in congenital glaucoma [11]; however, there have been few reports of such complications over a prolonged period [8].

Retinal detachment remains a source of poor visual prognosis in patients with congenital glaucoma with a reported incidence of 1% to 4.5% [6,8,11]. However, despite close follow-up and effective medical and surgical management of congenital glaucoma, ocular comorbidities including amblyopia play a large role in limiting final visual and anatomic outcomes. As seen in our case, despite a challenging but successful surgical outcome after RRD repair, amblyopia limited his visual potential to 20/70. Al Judaibi et al. conducted a retrospective comparative series of 10 eyes with congenital glaucoma and retinal detachment who underwent surgical repair with vitrectomy and endotamponade with or without scleral buckling. Their study demonstrated that factors such as amblyopia and glaucomatous optic nerve damage altered final visual outcomes with median visual acuity of HM [12]. Wiedemann et al. described five patients with congenital glaucoma who underwent vitrectomy with fluid/gas or fluid/silicone oil exchange for complicated retinal detachment and subretinal proliferation, but corneal decompensation and intraocular pressure elevation contributed to poor prognosis remained [11].

The presentation of retinal detachment in these patients is often delayed; advanced visual field changes from glaucomatous damage and suboptimal retinal examination from media opacities are often responsible. Retinal detachment repair outcomes remain poor in the setting of already reduced baseline vision [6,9,13]. In one study with a three-year follow-up period, re-detachment was noted in nine eyes of 33 eyes with total retinal detachment complicated by PVR in 21 eyes [13]. Cooling et al. reported successful re-attachment after RRD repair in only three of 13 eyes [6]. Corneal opacities and scarring related to both elevated intraocular pressure and corneal edema or corneal transplant rejection may preclude visualization of the posterior segment, thereby making it more difficult to monitor retinal pathology.

A variety of factors collectively pose a challenge in the management of retinal detachment repair, especially in buphthalmic eyes. Zhao et al. noted the degree of PVR as one of the two most significant factors affecting the prognosis for retinal detachment repair in congenital glaucoma [14]. This technical difficulty combined with poor visualization from potential media opacities allows for challenging surgical repair. The strong adherence of the vitreoretinal interface in these patients additionally makes the usual techniques used in PVD induction less successful. Our patient’s funduscopic examination demonstrated a subtotal retinal detachment with large nasal and inferior bullae with grade C PVR confirming the chronicity of the retinal detachment.

His poor baseline vision (congenitally buphthalmic eye) contributed to late detection of retinal detachment and influenced surgical success and visual outcome. Post-operative retinal re-detachment accompanied by hypotony maculopathy after silicone oil removal highlighted the challenging and complicated surgical course in these subsets of patients. Five thousand CS silicone oil does not emulsify (unlike 1000 CS) and was used for long-term tamponade. Although there currently is a lack of data regarding hypotony maculopathy in patients with congenital glaucoma, our findings are in concordance with other studies (globe survival remains the main surgical goal) [15].

## Conclusions

In summary, we emphasize the utilization of modern advances (perfluorocarbon liquids) in the successful repair of complex RRD in the setting of congenital glaucoma. In addition, 5,000 centistoke silicone oil (increased viscosity and stable non-emulsification) remains an important resource to prevent hypotonic maculopathy. Although successful retinal detachment repair may limit the final visual outcome (from associated ocular comorbidities and pre-existing amblyopia as seen in our patient), the risk of ensuing re-detachment, hypotony, and phthisis bulbi merits long-term endotamponade with silicone oil in an effort to achieve globe preservation and recovery of vision in this patient population.

## References

[1] Lim SH, Tran-Viet KN, Yanovitch TL (2013). CYP1B1, MYOC, and LTBP2 mutations in primary congenital glaucoma patients in the United States. Am J Ophthalmol.

[2] Cascella R, Strafella C, Germani C, Novelli G, Ricci F, Zampatti S, Giardina E (2015). The genetics and the genomics of primary congenital glaucoma. Biomed Res Int.

[3] Sarfarazi M, Stoilov I (2000). Molecular genetics of primary congenital glaucoma. Eye (Lond).

[4] Cronemberger S, Calixto N, Avellar Milhomens TG, Gama PO, Milhomens EG, Rolim H, Mendonça SC (2014). Effect of intraocular pressure control on central corneal thickness, horizontal corneal diameter, and axial length in primary congenital glaucoma. J AAPOS.

[5] Gupta V, Jha R, Srinivasan G, Dada T, Sihota R (2007). Ultrasound biomicroscopic characteristics of the anterior segment in primary congenital glaucoma. J AAPOS.

[6] Cooling RJ, Rice NS, Mcleod D (1980). Retinal detachment in congenital glaucoma. Br J Ophthalmol.

[7] Aziz A, Fakhoury O, Matonti F, Pieri E, Denis D (2015). Epidemiology and clinical characteristics of primary congenital glaucoma. (Article in French). J Fr Ophtalmol.

[8] Gupta S, Gogia V, Jose C, Chanana B, Bypareddy R, Kapoor KS, Gupta V (2016). Peripheral retinal degenerations and rhegmatogenous detachment in primary congenital glaucoma. Retina.

[9] Satofuka S, Imamura Y, Ishida S, Ozawa Y, Tsubota K, Inoue M (2008). Rhegmatogenous retinal detachment associated with primary congenital glaucoma. Int Ophthalmol.

[10] Knapp A (1916). Retinal detachment in hydrophthalmia. JAMA.

[11] Wiedemann P, Heimann K (1992). Retinal detachment in eyes with congenital glaucoma. Retina.

[12] Al-Harthi E, Al-Shahwan S, Al-Turkmani S, Khan AO (2007). Retinal detachment and congenital glaucoma. Ophthalmology.

[13] Al Judaibi RM, Al Hadlaq A, Ghazi NG (2014). Visual and anatomical outcomes in congenital glaucoma-related rhegmatogenous retinal detachment. Invest Ophthalmol Vis Sci.

[14] Zhao L, Wei Wei, B B (2014). Retinal detachment in congenital glaucoma. Acta Ophthalmologica.

[15] Ghoraba HH, El Gouhary SM, Ghali AA (2021). Pars plana vitrectomy with/without encircling scleral band for treatment of retinal detachment associated with buphthalmos. Int J Retina Vitreous.

